# Rational Use of Oxygen in COVID-19 Pandemic - Are We Doing Enough?

**DOI:** 10.31729/jnma.6479

**Published:** 2021-04-30

**Authors:** Gentle Sunder Shrestha, Ritesh Lamsal

**Affiliations:** 1 Department of Anaesthesiology, Tribhuvan University Teaching Hospital, Maharajgunj, Kathmandu, Nepal

**Keywords:** *COVID-19*, *oxygen*, *pandemics*

## Abstract

During the episodes of large case surge of COVID-19, the health care system of many nations have struggled, more so in nations with resource limitations. Recently, Nepal and the neighboring nation India are being hit hard by the pandemic. Management of patients with moderate and severe COVID-19 remains largely supportive, with oxygen therapy being the cornerstone of the management. Procurement, maintenance of oxygen supply system, coupled with avoiding misuse and wastage of oxygen is of paramount importance to better utilize the scarce resources amidst the peaks of a pandemic. Nepal needs to adopt policies to make best use of its oxygen stores and supplies with a collective effort from all stakeholders to save additional lives.

## INTRODUCTION

The second wave of COVID-19 pandemic has hit South-Asian countries hard, particularly India, where COVID-19 cases are increasing at an unprecedented rate.^1^ Nepal has also seen a surge of COVID-19 cases since the second week of April 2021.^2^ As the number of cases continues to rise exponentially, scarce hospital resources are already running thin, and critical care units are overburdened. As oxygen therapy is the cornerstone of management for patients with moderate and severe COVID-19, rational use of oxygen during the crisis cannot be overemphasized.

## CURRENT SCENARIO AND POSSIBLE SOLUTIONS

With a faltering healthcare system that had barely managed to grapple with the effects of the first COVID-19 wave, the overall situation appears grim with several pressing issues. The co-existence of multiple variants of SARS-CoV-2, inadequate vaccination infrastructure, deficient testing and tracing system, over-reliance on other countries for medical equipment, drugs and minimal stockpiling of essential medical supplies are concerning issues^3-6^ in the society likely due to pandemic fatigue, adherence to basic infection prevention measures like masking, avoiding mass gathering, physical distancing, and hand hygiene, are found wanting.

The evidence base for the use of therapeutics and supportive treatment in COVID-19 is increasing at a rapid pace, but several key areas have not been addressed adequately. One such issue is the rational use of oxygen, especially in countries like Nepal where indigenous production of oxygen is not adequate to meet the medical demand even in the best of times, let alone during the pandemic. Nearly 20% of patients with COVID-19 require hospitalization for oxygen therapy.^9^ According to one estimate, even before the pandemic, nine in ten hospitals in low- and middle- income countries (LMICs) lacked access to oxygen therapy, and only one-fifth of patients who needed medical oxygen received it.^10^ There are several media reports of a crippling shortage of oxygen in Indian hospitals; a similar situation in Nepali hospitals during the peak of the current wave is plausible. It is important to discuss current evidence on the principles and practice of oxygen therapy for hospitalized patients and the use oxygen in our patients rationally. It is equally important to focus on minimizing oxygen leaks while maximizing production by reinforcing medical gas supply systems in hospitals. But, are we doing enough to avert a possible 'oxygen crisis'?

It is common practice to provide oxygen to all sick patients in the hospital irrespective of their oxygenation status. This clinical practice is in part because of conventional medical teaching where a lot of emphasis is placed on highlighting the deleterious effects of hypoxia. Medical literature has abundant reports of complications of acute hypoxia such as fatal arrhythmia, increased risk of the systemic inflammatory response, and end-organ damage.^11–13^ However, recent evidence also suggests that liberal oxygen therapy in acutely ill adults is associated with increased in-hospital mortality.^14^ There is also a robust association between arterial hyperoxia and increased mortality in patients admitted to intensive care units.^15^ In acutely ill patients, there are strong recommendations to titrate oxygen therapy to target peripheral oxygen saturation (SpO_2_) no higher than 96% and to avoid starting oxygen therapy if SpO_2_ is already 93% or higher.^15^ These recommendations are also valid in patients admitted to the hospital with COVID-19. Often, less-is-more.

The rational use of oxygen is extremely relevant in the perioperative setup. There are recommendations for better adoption of neuraxial anesthesia and peripheral nerve blocks to obviate the need for general anesthesia, saving valuable resources, including oxygen, and also to decrease the risk of exposure to the infective aerosol.^16^ When providing general anesthesia, modern anesthesia ventilators with a circle system may not be readily available to utilize a low-flow anesthesia strategy in most resource-limited countries. Additive gaseous components like medical air are also seldom available. Consequently, open systems requiring high gas flows and a high fraction of inspired oxygen (FiO_2_) are common. For example, utilizing a circle system with a fresh gas flow of 1L per minute and a FiO_2_ of 0.3, only 36L of oxygen would be consumed in a 6-hour duration surgery.^17^ This requirement increases up to 792L when circle system and additive gaseous components are not available (fresh gas flow 6L per minute at a FiO_2_ of 0.5).^17^

It may not be possible to procure new equipment to reduce oxygen consumption during the ongoing crisis, but basic steps like routine maintenance to minimize circuit and pipeline leaks, stricter adherence to low-flow anesthesia whenever feasible, use of medical air if available, and using a low FiO_2_ if possible, seem to be the practical and realistic ways for rationalizing the use of oxygen. Although oxygen savings may not be significant, routine preoxygenation before induction of anesthesia, and use of high-flow, high-concentration of oxygen before and after tracheal extubation may not be required in all patients. Similarly, all patients in the immediate postoperative period do not need supplemental oxygen, unlike the common practice in many hospitals. Injudicious hyperoxemic therapy in surgical patients can increase the risk of absorption atelectasis, worsen ventilation-perfusion matching, reduce cardiac output, and delay the recognition of declining lung function in the post-anesthesia care unit.^18^

Even in patients admitted to the hospital with COVID-19-related acute respiratory failure, a recent expert consensus statement suggests maintaining a target SpO_2_ of only more than 90%.^19^ Another set of recommendations for managing acute respiratory failure in patients with COVID-19 in LMICs suggests maintaining SpO_2_ at 88 to 95%.^20^ Higher targets for SpO_2_ should only be used if continuous monitoring of SpO_2_ is not available.^20^ There are also several novel suggestions to improve oxygenation in critically ill COVID-19 patients. One recent study findings show that putting a surgical mask on top of a high-flow nasal cannula improves oxygenation in critically ill COVID-19 patients with hypoxemic respiratory failure.^21^

Another less-discussed issue is the general apathy of health officials in Nepal to bolster the indigenous oxygen production system, and to strengthen the procurement, storage, and distribution of medical oxygen, both before and after the first wave of COVID-19. There are glaring challenges, such as procurement hassles, unreliable power supply, poor maintenance standards, limited technical workforce, transportation difficulties, and lack of funds. Hopefully, the focus on oxygen sparked off by the current crisis will help hospitals develop the overall infrastructure for a sustained supply chain to help tackle the ongoing pandemic and beyond.

Management of patients with moderate to severe COVID-19 largely remains symptomatic. Oxygen therapy is the cornerstone of management. With the exceptions of a few pharmacological agents like corticosteroids and heparin, the role of other agents remains questionable. With only a small fraction of patients progressing to the point needing mechanical ventilation and critical care support, rational use of oxygen may be the single most important intervention to consider, while managing the case surges during the waves of COVID-19 pandemic.^22^

## WAYS FORWARD

Improving the clinical outcomes of patients admitted to the hospital with COVID-19 requires a concerted effort by all stakeholders. As resources are limited and the case-load in hospitals is rising fast, this is an opportune time to focus on the basics of medical care, including oxygen therapy. It is important to do the fundamentals right as we do not have adequate resources for the widespread adoption of resource-sapping protocols and expensive pharmacological agents that are common in high-income countries. It is equally crucial to find the right balance between hospital resources, patient safety, availability of trained manpower, and intensity of monitoring. Rational use of oxygen therapy is an important priority for hospitals in Nepal.
